# Trees shape the soil microbiome of a temperate agrosilvopastoral and syntropic agroforestry system

**DOI:** 10.1038/s41598-025-85556-4

**Published:** 2025-01-09

**Authors:** Anna Vaupel, Max Küsters, Julia Toups, Nadine Herwig, Benedikt Bösel, Lukas Beule

**Affiliations:** 1 Institute for Ecological Chemistry, Plant Analysis and Stored Product Protection, Julius Kühn Institute (JKI)–Federal Research Centre for Cultivated Plants, Berlin, Germany; 2 Finck Foundation gGmbH, Briesen (Mark), Germany

**Keywords:** Temperate agroforestry, Alley cropping agroforestry, Agrosilvopastoral system, Syntropic agriculture, Soil microbiome, Subsoil, Agroecology, Microbial communities, Biodiversity

## Abstract

Agroforestry systems are multifunctional land-use systems that promote soil life. Despite their large potential spatio-temporal complexity, the majority of studies that investigated soil organisms in temperate cropland agroforestry systems focused on rather non-complex systems. Here, we investigated the topsoil and subsoil microbiome of two complex and innovative alley cropping systems: an agrosilvopastoral system combining poplar trees, crops, and livestock and a syntropic agroforestry system combining 35 tree and shrub species with forage crops. Increasing soil depth resulted in a decline of bacterial and fungal richness and a community shift towards oligotrophic taxa in both agroforestry systems, which we attribute to resource-deprived conditions in subsoil. At each soil depth, the microbiome of the tree rows was compositionally distinct from the crop rows. We detected a shift towards beneficial microorganisms as well as a decline in putative phytopathogens under the trees as compared to the crop rows. Finally, based on our results on community dissimilarity, we found that compared to an open cropland without trees, spatial heterogeneity introduced by the tree rows in the agrosilvopastoral system translated into a compositionally less homogeneous soil microbiome, highlighting the potential of agroforestry to counteract the homogenization of the soil microbiome through agriculture.

**Supplementary Information:**

The online version contains supplementary material available at 10.1038/s41598-025-85556-4.

## Introduction

Agroforestry is an umbrella term that describes the fusion of agriculture and forestry on the same land that is practiced by millions of farmers around the globe and includes a large variety of transformative land-use systems. In the temperate zone, silvoarable systems that simultaneously grow woody perennials and crops are gaining increasing popularity^1,2^. Although the intentional spatial integration of tree rows into arable land (i.e. alley cropping) through cropland agroforestry can be associated with specific challenges (e.g. resource competition between trees and crops, labor-intensive management of trees, and lack of financial incentives), it offers numerous environmental benefits as compared to open croplands without trees^3^. For example, well-designed tree rows in alley cropping systems can effectively reduce wind speeds and the associated risk for soil erosion^4^ and improve microclimatic conditions^5^. Further environmental benefits include sequestration of C in the biomass of the trees as well as in soil^6^, promotion of biodiversity and related ecosystem services^7–9^, and improvement of soil health^10^. Another key benefit of trees in alley cropping systems is that they can reduce nutrient leaching as their deep rooting system can act as a “safety-net” for leached nutrients that are inaccessible to crops and would otherwise be lost from the system^11,12^. Although the “safety-net”-role of trees was first mentioned in the scientific literature decades ago^13^, this mechanism still requires further investigation^14,15^. Overall, integrating trees into annual cropping systems through alley cropping increases multifunctionality as compared to open croplands^3^.

Multiple ecosystem functions are mediated by above- and belowground biota including microorganisms in soil^16,17^. Soil microorganisms constitute a substantial part of global biodiversity^18^ and contribute to key soil functions such as nutrient cycling^19^. Therefore, their abundance, diversity, and functions are an integral^20^ but underrepresented^21^ part of soil health assessments. Temperate alley cropping agroforestry systems have consistently shown to promote the abundance, diversity, and functions of soil microorganisms as reviewed by Beule et al.^22^. As the integration of tree rows into arable land through agroforestry increases the complexity of the systems and therefore introduces spatial heterogeneity, sampling efforts in agroforestry systems are greater than in open croplands in order to capture spatial heterogeneity. This was already recognized over a quarter of a century ago^23^ and found implementation in the sampling designs of numerous recent studies on soil microorganisms in alley cropping agroforestry systems as reviewed recently^22^. Commonly, spatial gradients from the trees into the crops as well as different soil depths are investigated. Realizing such a study design, several studies reported that soil microbial population size and activity were not just promoted under the trees but that the beneficial effects of the trees gradually extend into the crop rows^24,25^. In addition, Beule et al.^14^ found that microbial communities in subsoil were stronger promoted by trees in agroforestry systems than those in topsoil. The extent to which beneficial effects of tree rows on soil microbial communities expand into the crop rows of agroforestry systems is hypothesized to be mainly depending on the distribution of below- (rhizodeposits and root litter) and aboveground tree litter inputs (leaf litter)^22^.

Despite an ever-growing body of literature on soil microorganisms in temperate cropland agroforestry systems, most research efforts focused on conventionally farmed alley cropping systems that combine a single tree species (mostly poplar or walnut) with a rather narrow crop rotation of annual crops (mostly small-grain cereals or maize). This ignores the large diversity in spatial and temporal configuration and management of agroforestry systems in the temperate zone. For instance, agrosilvopastoral systems, which integrate trees with crops and livestock, are highly understudied although such integrated crop-livestock systems offer several benefits^26^. A prominent example for such benefits is the grazing of cover crops grown between cash crops, which offers economic returns without compromising soil health^27–29^. Grazing is also known to influence microbial communities^30^. For instance, grazing of cover crops has recently been shown to increase microbial biomass^31^. Furthermore, carefully selected tree species can benefit animal welfare through, *inter alia*, provision of shelter, improved digestion, and enhanced parasite control^32^. Similar to agrosilvopastoral systems, syntropic agroforestry systems, in which complex tree components are designed to mimic natural succession and stratification in order to maximize synergies among plants, are not yet in the focus of research. This is surprising given that syntropic agriculture, also referred to as dynamic or successional agroforestry, is gaining increasing popularity and is perceived as an innovative approach to agroecology^33^. Syntropic agroforestry involves a complex and diverse planting scheme that aims to mimic a forest ecosystem to create a resilient, self-sufficient, and constantly evolving system that is supposed to produce long-term yields without external inputs (e.g. fertilizer and irrigation). Due to greater plant diversity and density compared to open croplands, syntropic agroforestry systems can be expected to have a strong impact on soil microbial communities, especially through increased quality and quantity of rhizodeposition and leaf litter inputs^34–36^. Finally, organic agriculture, which –unlike in conventional agriculture– abandons synthetic fertilizers and pesticides, has shown a significant increase during the last 25 years^37^. However, studies investigating soil microbial communities in organically farmed temperate cropland agroforestry systems are scarce. Recently, Rosati et al.^38^ collected evidence that despite challenges, the adoption of agroforestry in organic agriculture can result in multiple environmental benefits. The authors further argued that organic farmers are expected to be more open to the adoption of agroforestry as compared to their conventional counterparts^38^. As highlighted above, despite their large potential spatial and temporal complexity, the majority of studies that investigated soil life in temperate agroforestry systems is at the lower end of the spectrum in terms of system complexity.

Our study aims to investigate the soil microbiome in topsoil and subsoil of two temperate alley cropping agroforestry systems (an agrosilvopastoral and a syntropic system) under organic farming in Germany. We determined the population size of soil archaea, bacteria, and fungi using real-time PCR and assess the diversity and community composition of soil bacteria and fungi by amplicon sequencing. We hypothesized that (i) microbial population size decreases with soil depth due to resource limitations in deeper soil layers. Furthermore, we hypothesized that (ii) microbial populations size increases with decreasing distance to the trees due to increased tree litter inputs (rhizodeposition and leaf litter) in close proximity to the trees. We further expected that (iii) microbial communities within topsoil have greater community similarity than those within subsoil as a result of homogenization of topsoil communities due to soil management and that (iv) tree rows promote beneficial microorganisms rather than putative plant pathogens.

## Materials and methods

### Study sites

Our study was conducted at (1) a paired agrosilvopastoral alley cropping system (28.4 ha, 52°22’37.70"N, 14°17’25.29"E, m.a.m.s.l.: 65 m) and open cropland system and (2) a syntropic alley cropping system (3.2 ha, 52°22’58.24"N, 14°17’12.52"E, m.a.m.s.l.: 66 m). Both, the agrosilvopastoral and syntropic alley cropping system, were under agricultural use for at least 40 years prior to conversion of open cropland to agroforestry in 2019. Both study sites were under organic farming since 2004 and located on Eutric Retisol and Geoabruptic Luvisol soils near Briesen (Mark), in the federal state of Brandenburg, Germany (mean annual air temperature 1991–2020: 9.4 °C; mean annual precipitation 1991–2020: 545.6 mm). Brandenburg is characterised by its sandy and sandy loamy soils that have low water-holding capacity^39^. In combination with low annual precipitation mostly below 600 mm and a decreasing trend in available soil water^40^, Brandenburg is among the most vulnerable regions to climate change in Germany^41^.

### Agrosilvopastoral alley cropping system

The agrosilvopastoral alley cropping system comprised six 4-m wide rows of trees that were planted in North-South orientation and alternated with 36-m wide rows of arable crops. The tree rows of the agrosilvopastoral system covered 8.7% of the total area of the system and consisted of 30-m long segments of different tree species for biomass production. The majority of trees were poplar (*Populus maximowiczii* × *P*. *trichocarpa* (clone Androscoggin), *P*. *trichocarpa* (clone Columbia River and Muhle Larsen), and *P*. x *euramericana* (clone Jacometti 78 B)), willow (*Salix purpurea* and *S. caprea*), and alder trees (*Alnus incana*). Each tree row comprised two tree lines that were planted by hand using a dibble bar. Prior to planting, a planting furrow was created for the trees utilizing a forest plough and trees were planted at 1 m distance within each tree line. Soil sampling was conducted in segments of poplar clones Columbia River, Jacometti 78 B, and Muhle Larsen. Within these segments, the poplar clones were alternated with an alder tree every four trees. At the time of sampling, the aboveground biomass of the four-year-old trees has not been harvested yet. The agrosilvopastoral system was spatially paired with an adjacent open cropland system that served as a reference land use without trees. The crop rotation of the crop rows of the agrosilvopastoral system was identical to that of the adjacent open cropland since 2022. In 2022, winter rye (*Secale cereale*) with an undersown grass-clover mixture was cultivated. From October 2022 until March 2023, the undersown grass-clover mixture at both the agrosilvopastoral system and the open cropland were grazed by a beef cattle (*Bos taurus*) herd of 140 individuals of Angus and Salers breed. The herd was managed under year-round grazing with grazing on cover crops and undersown crops and additional hay and silage feeding during winter. After the herd was moved from the study site, the crop row of the agrosilvopastoral system and the open cropland did not receive any fertilizer prior to drilling of summer oat (*Avena sativa*) in spring 2023. The crop rows of the agrosilvopastoral system as well as the open cropland were managed under reduced tillage since the establishment of the agroforestry system.

### Syntropic alley cropping system

**Fig. 1:**
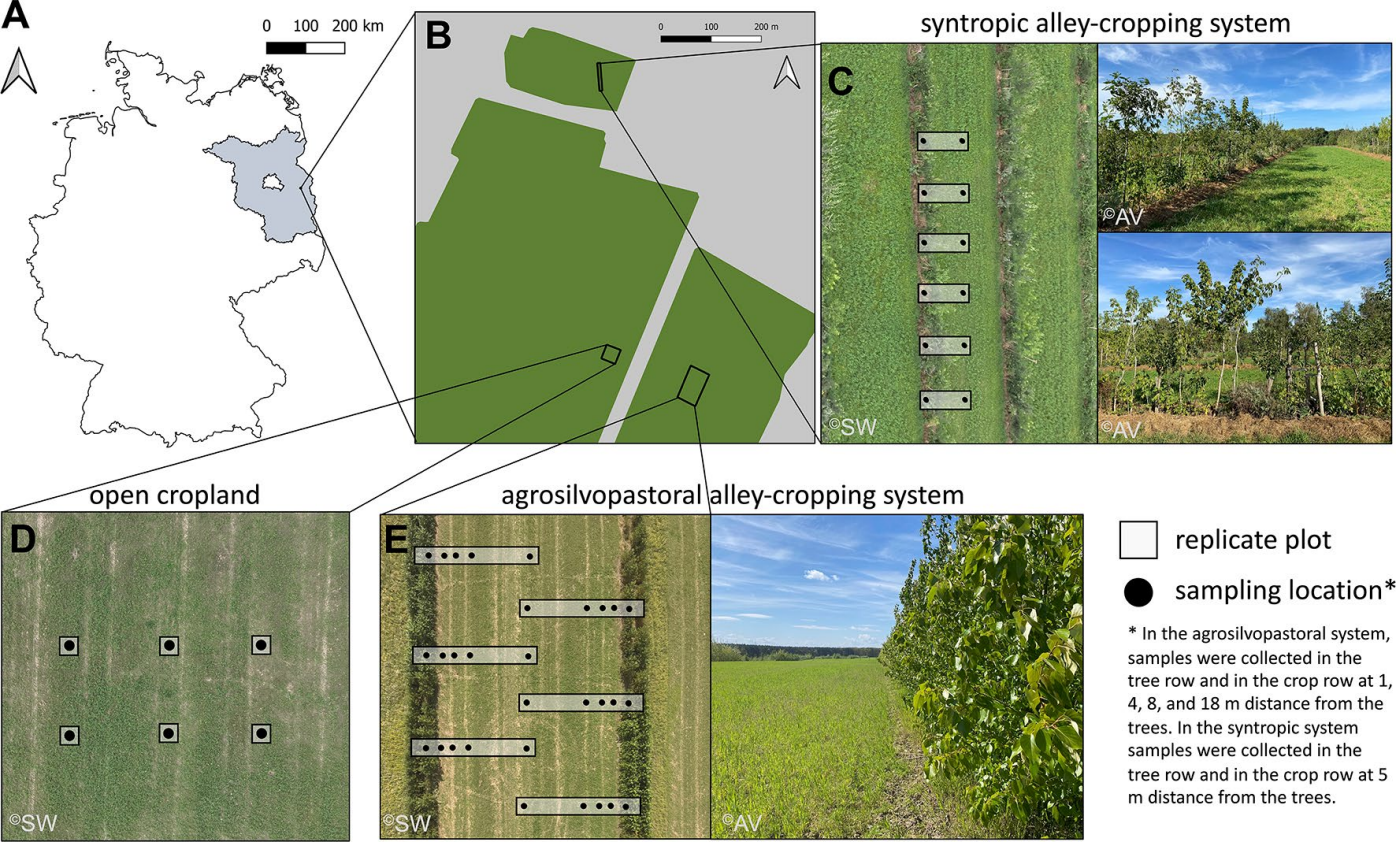
Location of the study sites near Alt Madlitz in the federal state of Brandenburg (highlighted in grey), Germany (**A**, **B**). Study design and photos of the syntropic alley cropping system (**C**), the open cropland (**D**), and the agrosilvopastoral and alley cropping system (**E**). Photo credit: Sam Waltl (Finck Foundation) and Anna Vaupel (JKI). Maps were created using Natural Earth (naturalearthdata.com) and QGIS version 3.4.4-Madeira.

The syntropic alley cropping system comprised 19 hand-planted 1 m-wide rows of trees in North-South orientation that were alternated with 10 m-wide rows of forage crops (grass-alfalfa mixture). The tree rows covered 9.2% of the total area of the system and comprised a single line of trees bordered by a layer of mulch comprising wood chips and cut biomass of the forage crops (Fig. 1). The system is specialized on fruit and nut production and applies the principles of syntropic agroforestry. The system can be classified as a successional agroforestry system due to the integration of more than two stratification levels (‘strata’) of trees and shrubs, the plant selection which is based on ecological succession, and the dynamic management approach^42^.

Within the tree rows of the syntropic system, fruit and nut trees were the main tree crops, which were densely grown along with timber trees and ‘support species’ that are supposed to promote ecosystem functions. The selection of plant species as well as the spatial arrangement of trees and shrubs followed their position in stratification and ecological succession. As support species, the system included undemanding plants such as herbs, sea buckthorn, and blackberries as well as fast-growing pioneer tree species such as poplar, birch, and wild cherry. As succession proceeds, pioneer trees will be superseded by fruit, nut, and timber trees, and finally by oak and maple trees, which were grown from seeds and reach maturity after several decades. Plants are further cultivated at four strata according to their individual light requirements. For example, ground-covering herbaceous plants such as mugwort, mallow, tarragon, and comfrey grow at the lowest strata, followed by sea buckthorn or berries at the second lowest strata, followed by fruit and nut trees, and finally pioneer trees at the highest strata. The management of the tree rows included manual weeding, dynamic pruning of timber and support species to maintain stratification as well as fruit and nut trees to ensure yield production, and adding the pruned biomass to the layer of mulch bordering the tree rows. The forage crop rows were cut up to four times a year for hay production. Forage crop cuttings within 2 m distance to the tree rows were not used for hay production and swathed onto the layer of mulch. Overall, the syntropic system comprised 35 tree and shrub species of different varieties as well as 20 herbaceous plant species.

Soil sampling was conducted in the fourth tree row from the eastern end of the system (Fig. 1) with plums and pears as main fruit crops. Within the tree row, 18 plum varieties were spaced at a distance of 2 m and six pear varieties were spaced at a distance of 10 m. Between plum and pear trees, birch, poplar, and wild cherry trees were planted that were in turn alternated with blackberries and diverse herbaceous plants as ground cover.

### Soil sampling and general soil properties

At each sampling location, soil samples were collected in topsoil (0–30 cm) and subsoil (30–60 cm) on 20.09.2023 using stainless steel augers. Furthermore, three soil cores per soil depth were collected at each sampling location (technical replicates), transferred into a sterile polyethylene bag, and thoroughly homogenized in the field to obtain a composite sample per sampling location (biological replicate). To account for spatial heterogeneity introduced by the trees^24,25^, soil samples within the agroforestry system were collected at different sampling locations along six transects spanning from the centre of the tree row into the centre of the crop row. The transects were delineated orthogonal to the north-south orientation of the tree rows and covered the tree row as well as 1, 4, 8, and 18 m distance (centre of the crop row) from the trees into the crop row. Furthermore, an adjacent open cropland cultivated with identical crops and under identical management practices as the crop row of the agroforestry system served as a reference land use. In the open cropland, soil samples were collected at six sampling locations. Thus, we collected 72 soil samples in the agrosilvopastoral alley cropping system (6 sampling locations (tree row, 1, 4, 8, and 18 m crop row and open cropland) × 6 replicates × 2 soil depths = 72 samples). In contrast to the agrosilvopastoral alley cropping system, crop rows in the syntropic alley cropping system were only 10 m wide. Therefore, soil samples in the syntropic alley cropping system were only collected in the tree row as well as in the centre of the crop rows at 5 m distance from the trees using six replicates. For this system, an open cropland serving as a reference land use was unavailable. Thus, we collected 24 soil samples in the syntropic alley cropping system (2 sampling locations (tree row, 5 m crop row) × 6 replicates × 2 soil depths = 24 samples). From each of the 96 soil samples (72 from the agrosilvopastoral and 24 from the syntropic system), an aliquot of approx. 50 g fresh soil was transferred into a sterile 50-ml Falcon tube and frozen at −20 °C in the field for molecular analyses. The remaining soil was used for the analyses of general soil properties.

Soil pH, SOC, and total N were determined from air-dried, sieved (< 2 mm) soil. Soil pH was determined in 0.01 M CaCl_2_ at a ratio of 1:2.5 (soil: CaCl_2_(w/v)). For SOC and total N, air-dried and sieved soil was finely ground and subjected to acid fumigation^43^ to remove inorganic C. Following acid fumigation, SOC and total N were determined utilizing a CNS elemental analyzer (Vario EL Cube, Elementar, Germany).

### Soil DNA extraction

Upon arrival at the laboratory, samples were stored at −20°C until freeze-drying for 72 h. Freeze-dried soil was finely ground using a vortexer as described earlier^44^. Soil DNA was extracted using an extraction protocol tailored to subsoil samples as per Guerra et al.^45^. Briefly, 200 mg freeze-dried and finely ground soil were suspended in 250 µL phosphate lysis buffer (1 M phosphate buffer with 0.5% SDS (w/v)) and incubated at 65 °C for 10 min. The suspension was centrifuged, the supernatant was transferred into a new reaction tube, diluted 1:10 (v/v) in double-distilled H_2_O (ddH_2_O) and extracted twice with chloroform-isoamyl alcohol (24:1 (v/v)). Following this, DNA was allowed to precipitate for 15 min at room temperature under the presence of 30% (w/v) polyethylene glycol (PEG 6000) and 5 M NaCl (2:1 (v/v)). Precipitated DNA was pelleted using centrifugation and obtained pellets were washed twice with 80% (v/v) EtOH prior to drying the pellets in a vacuum centrifuge. Dried pellets were resuspended in 50 µL of 1× TE buffer (10 mM Tris, 1 mM EDTA, adjusted to pH 8.0 with HCl) and incubated for 42 °C for 2 h to facilitate resuspension of DNA. The quantity and quality of the extracted DNA was visually checked on 1% (w/v) agarose gels stained with SYBR Green I solution (Thermo Fisher Scientific GmbH, Dreieich, Germany).

### Real-time PCR assays for soil archaea, bacteria, and fungi

Soil archaea, bacteria, and fungi were quantified using real-time PCR using the primer sets ARC787F/ARC1059R^46^, Bac349F/Bac806R^47^ and ITS3/ITS4^48,49^, respectively. To overcome PCR inhibition, DNA extracts were diluted 1:50 (v/v) in ddH_2_O prior to PCR^45^. PCR reactions were performed in a 384-Well real-time PCR cycler (qTOWER3 84 G, Analytik Jena GmbH + Co. KG, Jena, Germany) in 5-µL reaction volumes. The reaction volumes were comprised of 4 µL master mix (ddH_2_O, primer (0.3, 0.5, and 0.3 µM of each primer for archaea, bacteria, and fungi, respectively), and 2.5 µL 1× Luna^®^ Universal qPCR Master Mix (New England Biolabs, Beverly, Massachusetts, USA)) and 1 µL template DNA or ddH_2_O for a negative control. The thermocycling conditions were as follows: initial denaturation at 95 °C for 5 min followed by 40 cycles of denaturation at 95 °C for 40 s, annealing at 60, 53, and 58 °C for 30 s for archaea, bacteria, and fungi, respectively, and elongation at 68 °C for 30 (archaea) or 60 s (bacteria and fungi). Final elongation was carried out at 68 °C for 5 min. Following amplification, melting curves were generated by step-wise increasing the temperature from 65 °C to 95° at a rate of 1 °C per step under continuous fluorescence measurement.

### Amplicon sequencing of soil bacteria and fungi

Sequencing libraries for soil bacteria and fungi were constructed using primer pairs 341F/785R^50^and ITS1-F_KYO2/ITS86R^51,52^, respectively. PCR was performed using an Eppendorf Mastercycler EP Gradient S thermocycler (Eppendorf, Hamburg, Germany) in 25-µL reaction volumes containing 18.75 µL master mix and 6.25 µL template DNA or ddH_2_O for a negative control. The master mix contained ddH_2_O, buffer (10 mM Tris–HCl, 50 mM KCl, 2.0 mM MgCl_2_, pH 8.3 at 25°C), 100 µM of each deoxynucleoside triphosphate (New England Biolabs, Beverly, Massachusetts, USA), 0.5 µM of each primer, 1 mg mL^−1^ bovine serum albumin, and 0.03 u µL^−1^ Hot Start *Taq* DNA Polymerase (New England Biolabs, Beverly, Massachusetts, USA). As per Bednar et al.^53^, each primer was a mixture of the primer with (50%) and without (50%) Illumina TruSeq 5’-end adapters. We used a touch-up thermocycling protocol^54^ to amplify bacteria and fungi. The thermocycling conditions were as follows: initial denaturation at 95 °C for 2 min, 3 touch-up cycles (denaturation: 95 °C for 20 s, annealing: 50 °C for 30 s, and elongation: 68 °C for 60 s), 25 and 27 cycles (denaturation: 95 °C for 20 s, annealing: 58 °C for 30 s, and elongation: 68 °C for 60 s) for bacteria and fungi, respectively. Final elongation was carried out at 68 °C for 5 min. Amplicons were screened on 1.7% (w/v) agarose gels as described above. Sequencing libraries were subjected to a second amplification with standard i7- and i5- sequencing adapters, multiplexed, and sequenced on an Illumina MiSeq (V3 chemistry, 2 × 300 bp) (Illumina, Inc., San Diego, CA, USA) at the facilities of LGC Genomics (Berlin, Germany). Amplicon sequencing data have been deposited at NCBI’s Sequence Read Archive (BioProject numbers PRJNA1142703 for bacteria and PRJNA1142770 for fungi).

### Processing of amplicon sequencing data and statistical analysis

Obtained sequence reads were processed as detailed in Bednar et al.^53^. First, data were demultiplexed utilizing Illumina’s bcl2fast version 2.20 (Illumina, San Diego, CA, USA). Demultiplexed data were cleaned from one-sided and conflicting barcodes as well as barcodes containing more than two mismatches. Furthermore, sequencing adapter and primer sequences as well as sequence reads with < 100 bp were removed. The cleaned sequence reads were processed in QIIME 2 (version 2022.2^55^). We used DADA2^56^ to quality filter, merge, and remove chimeras and singletons from the data. Amplicon sequencing variants (ASVs) were taxonomically classified against the SILVA ribosomal RNA gene database release 138^57^ for bacteria and UNITE database version 9.0^58^ for fungi. We utilized a scikit-learn Naive Bayes machine-learning classifier (‘q2-fit-classifier-naive-bayes’ and ‘q2- classify-sklearn’ plugin) in the ‘balanced’ configuration ([7,7]; 0.7 for bacteria and [6,6]; 0.96 for fungi as suggested by Bokulich et al.^59^) for classification. Non-bacterial and non-fungal sequence reads were removed from the bacterial and fungal data set, respectively.

Downstream analyses of the obtained ASV tables were performed in R version 4.4.1^60^ using the ‘microeco’ R package version 1.7.1^61^. ASV tables were normalized to account for uneven sequencing depth using scaling with ranked subsampling (SRS)^62^ using the ‘SRS’ R package version 0.2.3^63^ as implemented in ‘microeco’. The bacterial ASV table was normalized to 30,098 sequence reads. The fungal ASV table was normalized to 55,287 sequence reads. Alpha diversity indices (Shannon diversity, Chao1 index, and Pielou’s evenness) and Bray-Curtis dissimilarity were calculated in ‘microeco’. Differences in microbial community composition among different soil depths and sampling locations within each agroforestry system were identified with Permutational Multivariate Analysis of Variance (PERMANOVA) based on the ‘adonis 2’-function of the R package ‘vegan’. Prior to using PERMANOVA, permutation tests for homogeneity of multivariate dispersions were performed using the ‘permdisp’ function of the ‘vegan’ package. Differences in Bray-Curtis dissimilarities were identified using Wilcoxon Rank Sum test for pairwise comparisons (i.e. soil depth or sampling locations within the syntropic system) and Kruskal-Wallis Rank Sum test followed by Dunn’s multiple comparisons test for more than two groups (i.e. the six sampling locations within the paired agrosilvopastoral and open cropland system). Differences in alpha diversity indices, microbial population sizes, and general soil properties between different soil depths and sampling locations within each agroforestry system were identified using one-way ANOVA followed by Tukey HSD test or Student’s *t*-test for pairwise comparisons (i.e. soil depth or sampling locations within the syntropic system). For the analysis of different soil depths, linear mixed-effect models were used with soil depth as a fixed effect and sampling location as random effect. Before performing ANOVA, all data were tested and visually checked to meet the criteria of normality of residuals (Shapiro-Wilk test) and homogeneity of variance (Levene’s test). Data not meeting the criteria were either log- (soil microbial population sizes) or square root-transformed (alpha diversity indices). Differential abundance analysis was performed at all taxonomic levels utilizing linear models for differential abundance analysis (LinDA)^64^. For differential abundance analysis of more than two groups (i.e. among the six sampling locations within the paired agrosilvopastoral system and open cropland), tree row was set as reference group. For differential abundance analysis of different soil depths, linear mixed-effect models were used with soil depth as a fixed effect and sampling location as random effect. For all tests, statistical significance was considered at *p* < 0.05.

## Results

### General soil properties

In both agroforestry systems (agrosilvopastoral and syntropic alley cropping system), SOC and total N were greater in topsoil than subsoil (*p* < 0.001) (Figure S1 A, B, C, D). In topsoil of the paired agrosilvopastoral and open cropland system, few sporadic differences in SOC and total N among sampling locations were found with slightly lower SOC (*p* < 0.0021) and total N (*p* < 0.0027) in the open cropland as compared to the crop row of the agrosilvopastoral system (Figure S1 A, C). In contrast, no statistically significant differences in SOC and total N among sampling locations were detected in subsoil. Soil pH remained unaffected by the agrosilvopastoral system at both soil depths. In the syntropic agroforestry systems, SOC (*p* < 0.001) and total N (*p* < 0.016) were greater under the trees as compared to the crop row at both soil depths (Figure S1 A, B, C, D). Furthermore, the tree row of the syntropic system had a higher soil pH than its corresponding crop row in topsoil (*p* < 0.001) (Figure S1 E).

### Population size of soil bacteria, fungi, and archaea

**Fig. 2:**
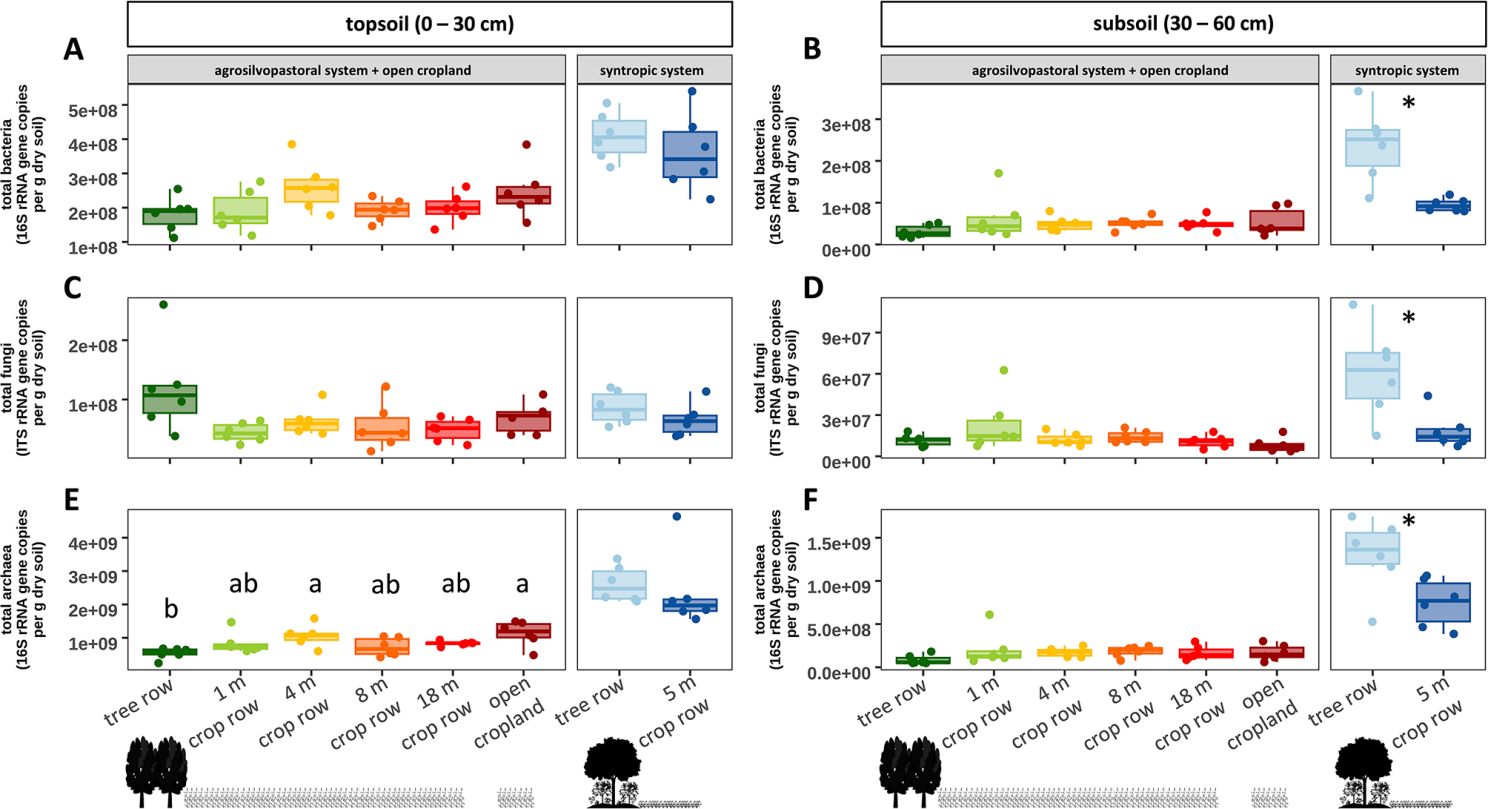
Soil microbial population size of total bacteria (**A**, **B**), fungi (**C**, **D**) and archaea (**E**, **F**) in topsoil and subsoil of an agrosilvopastoral and syntropic alley cropping system near Alt Madlitz, Germany (*n* = 6). Dots indicate individual data points. Different letters indicate statistically significant differences among the six sampling locations within the agrosilvopastoral alley cropping system (ANOVA followed by Tukey’s HSD test; *p* ≤ 0.05). Asterisks indicate statistically significant differences between the two sampling locations within the syntropic alley cropping system (Student’s *t*-test; *p* ≤ 0.05). Absence of letters or asterisks indicates no statistically significant differences among sampling locations (*p* > 0.05). Icons were created with BioRender.com.

Soil microbial population size was overall greater in topsoil than subsoil for bacteria, fungi, and archaea (*p* < 0.001). Differences between the tree row and the crop row of the syntropic system were more pronounced in subsoil than in topsoil, where soil microbial population sizes of bacteria, fungi and archaea were greater under the trees than in the centre of the crop row (*p* < 0.001, *p* = 0.011, and *p* = 0.048, respectively) (Fig. 2B, D, F). In the agrosilvopastoral system, soil microbial population size did not differ between different sampling locations (tree row, 1, 4, 8, and 18 m crop row and open cropland) regardless of soil depth, except for archaea in topsoil, which showed slightly lower gene copy numbers under the trees than at 4 m distance in the crop row (*p* = 0.015) and the open cropland (*p* = 0.008) (Fig. 2E).

### Alpha diversity of soil bacteria and fungi

**Fig. 3:**
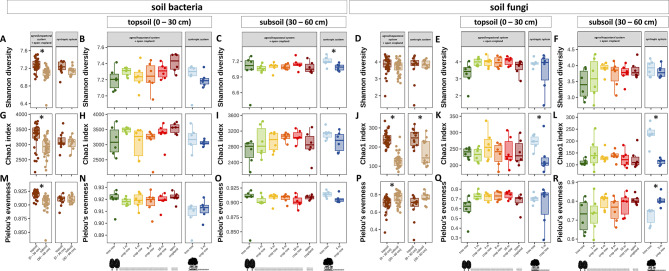
Alpha diversity indices (Shannon diversity (**A**, **B**, **C**,** D**,** E**,** F**), Chao1 index (**G**,** H**,** I**,** J**,** K**,** L**), and Pielou’s evenness (**M**,** N**,** O**,** P**,** Q**,** R**)) of soil bacterial and fungal amplicon sequencing variants (ASVs) in topsoil and subsoil of an agrosilvopastoral and syntropic alley cropping system near Alt Madlitz, Germany (*n* = 6). Dots indicate individual data points. Different letters indicate statistically significant differences among the six sampling locations within the agrosilvopastoral alley cropping system (ANOVA followed by Tukey’s HSD test; *p* ≤ 0.05). Asterisks indicate statistically significant differences between the two soil depths across sampling locations (linear mixed effect models; *p* ≤ 0.05) as well as the two sampling locations within the syntropic alley cropping system (Student’s *t*-test; *p* ≤ 0.05). Absence of letters or asterisks indicates no statistically significant differences between soil depths or sampling locations (*p* > 0.05). Icons were created with BioRender.com.

Alpha diversity indices (Shannon diversity, Chao1 index, and Pielou’s evenness) of soil bacterial ASVs were greater in topsoil than subsoil in the agrosilvopastoral system (*p* ≤ 0.001) but did not differ between soil depths in the syntropic system (Fig. 3A, G, M). In the agrosilvopastoral system, bacterial alpha diversity indices showed no statistically significant differences among sampling location in both topsoil and subsoil (Fig. 3B, C, H, I, N, O). In subsoil of the syntropic system, bacterial Shannon diversity was greater under the trees than in the centre of the crop row (*p* = 0.034) (Fig. 3C). Shannon diversity of soil fungi remained unaffected by soil depth and sampling location in both agroforestry systems (Fig. 3D, E, F). However, fungal ASV richness (Chao1 index) was greater in topsoil then subsoil in both systems (*p* < 0.001) (Fig. 3J) as well as in the tree row as compared to the centre of the crop row within the syntropic system at both soil depths (*p* = 0.046 and *p* < 0.001 for topsoil and subsoil, respectively) (Fig. 3K, L). Evenness of fungal ASVs (Pielou’s evenness) was greater in subsoil than topsoil of the agrosilvopastoral system (*p* < 0.001) (Fig. 3P) as well as in the centre of the crop row than under the trees in subsoil of the syntropic system (*p* = 0.004) (Fig. 3R).

### Community composition of soil bacteria and fungi

**Fig. 4:**
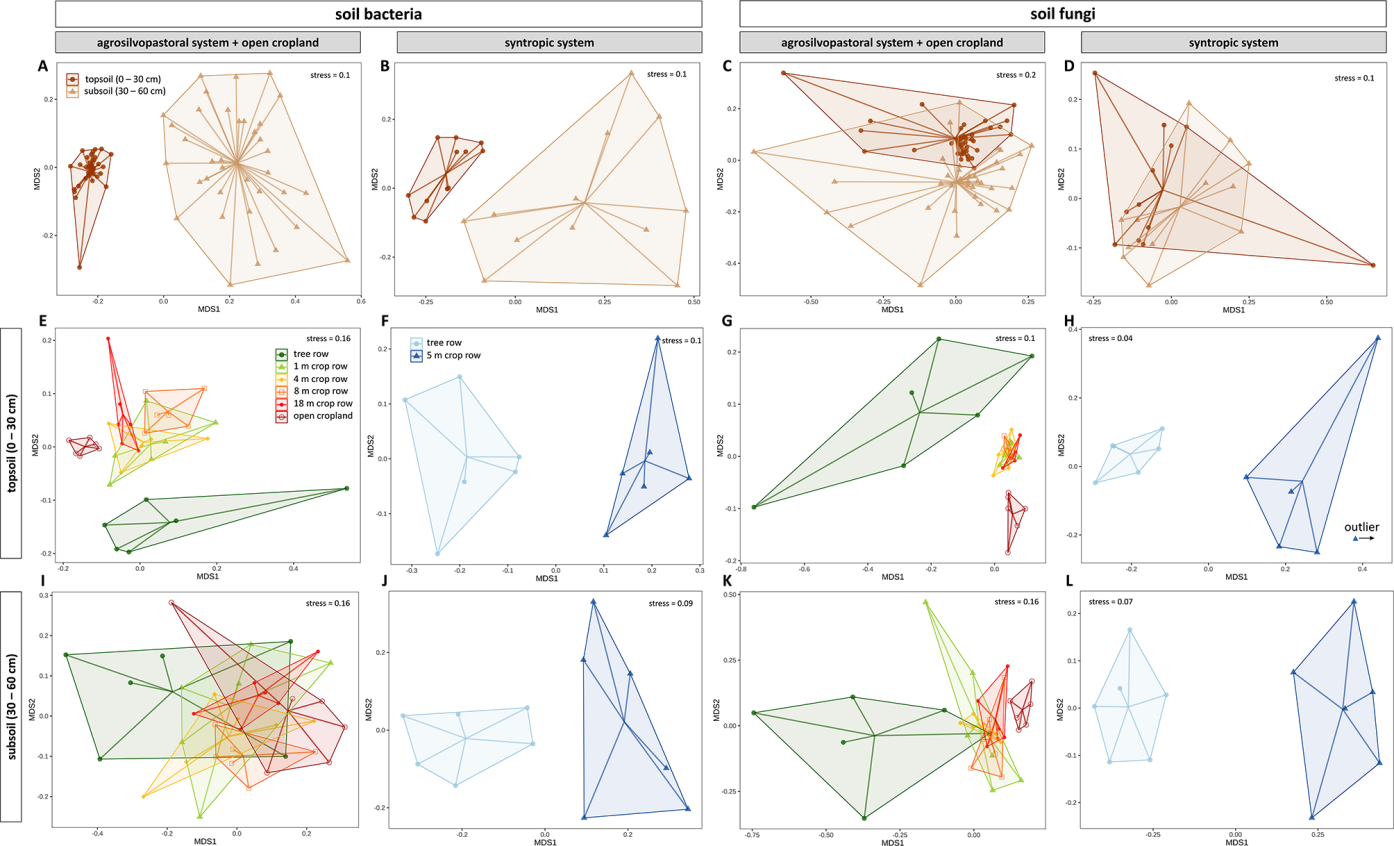
Non-metric multidimensional scaling (NMDS) of Bray–Curtis dissimilarities of bacterial and fungal communities among sampling depths (**A**,** B**,** C**,** D**) of an agrosilvopastoral (*n* = 36) and syntropic alley cropping system (*n* = 12) as well as among sampling locations in topsoil (**E**,** F**,** G**,** H**) and subsoil (**I**,** J**,** K**,** L**) of both systems (*n* = 6) near Alt Madlitz, Germany. Dots indicate individual data points.

**Fig. 5:**
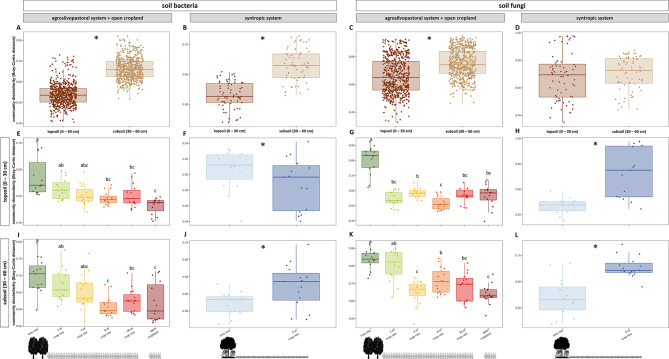
Community dissimilarity of soil bacteria and fungi within each soil depth (**A**,** B**,** C**,** D**) and at each sampling location in topsoil (**E**,** F**,** G**,** H**) and subsoil (**I**,** J**,** K**,** L**) of an agrosilvopastoral and syntropic alley cropping system near Alt Madlitz, Germany. Community dissimilarity was assessed using pairwise Bray-Curtis distances between individual samples. Dots indicate pairwise Bray-Curtis distances between samples. Different letters indicate statistically significant differences among the six sampling locations within the agrosilvopastoral alley cropping system (Kruskal-Wallis test followed by Dunn’s test; *p* ≤ 0.05). Asterisks indicate statistically significant differences between the two soil depths across sampling locations (linear mixed effect models; *p* ≤ 0.05) as well as the two sampling locations within the syntropic alley cropping system (Wilcoxon Rank Sum test; *p* ≤ 0.05). Absence of letters or asterisks indicates no statistically significant differences between soil depths or sampling locations (*p* > 0.05). Icons were created with BioRender.com.

Across all samples, *Acidobacteria* (20.68 ± 2.91%), *Proteobacteria* (15.77 ± 1.87%), and *Actinobacteria* (14.84 ± 3.51%) were the most abundant bacterial phyla, whereas fungal communities were dominated by the phyla *Ascomycota* (40.52 ± 13.10%), *Basidiomycota* (20.44 ± 17.79%), and *Mortierellomycota* (9.59 ± 6.01%). In both agroforestry systems, soil bacterial community composition differed between topsoil and subsoil (*p* < 0.001) (supplementary Table S1). The compositional differences in soil bacterial communities between soil depths were also reflected by the clustering of the data in the NMDS plot (Fig. 4, A, B) as well as by the number of unique and shared ASVs between topsoil and subsoil (Figure S2, A, B). Within each agroforestry system, topsoil and subsoil each had at least twice as much unique bacterial ASVs than shared ones across depths; however, the shared ASVs across depths accounted for approximately three quarters of total ASV counts (Figure S2 A, B). The composition of the soil bacterial community in topsoil of the agrosilvopastoral systems was strongly affected by the sampling location as the tree row, the different distances from the trees within the crop row, and the open cropland all harboured compositionally distinct communities (supplementary Table S2, Fig. 4E). Similarly, community composition of bacteria in the syntropic system differed among the tree and crop row at both soil depths (supplementary Table S3, Fig. 4F, J). Community dissimilarity of soil bacteria was greater in subsoil than topsoil across sampling locations in both agroforestry systems (*p* < 0.001) (Fig. 5A, B). Within each soil depth of the agrosilvopastoral system, community dissimilarity of soil bacteria gradually decreased with increasing distance from the trees (Fig. 5E, I). In subsoil of the syntropic system, bacterial community dissimilarity was greater in the crop row than in the tree row (*p* = 0.016) (Fig. 5J).

Soil depth was a strong driver of the fungal community composition in the agrosilvopastoral but not in the syntropic system (supplementary Table S5, Fig. 4C, D). In both agroforestry systems, topsoil harboured more unique fungal ASVs than subsoil whereas the number of ASVs shared between both soil depths was greater than in subsoil but lower than in topsoil and accounted for more than 90% of total ASV counts (Figure S2, C, D). At both soil depths, community composition of soil fungi in the agrosilvopastoral was distinct among the tree row, the crop row, and the open cropland (supplementary Table S6). Likewise, in the syntropic system, the composition of the fungal community was different between the tree and crop row at both topsoil and subsoil (supplementary Table S7, Fig. 4H, L). Soil fungi showed greater community dissimilarity in subsoil than in topsoil across all sampling locations of the agrosilvopastoral system (*p* < 0.001) (Fig. 5C), whereas no differences were found between soil depths in the syntropic system (*p* = 0.191) (Fig. 5D). Fungal community dissimilarity was greater under the trees than in the crop rows of the agrosilvopastoral system and the adjacent open cropland in topsoil (Fig. 5G). While no decline in community dissimilarity with increasing distance from the trees was observed in topsoil, a similar decline as for bacteria was found for soil fungi in subsoil of the agrosilvopastoral system (Fig. 5K). At both soil depths, community dissimilarity of soil fungi was greater in the crop row than in the tree row of the syntropic system (*p* < 0.001) (Fig. 5, H, L).

### Differential abundance analysis

**Fig. 6:**
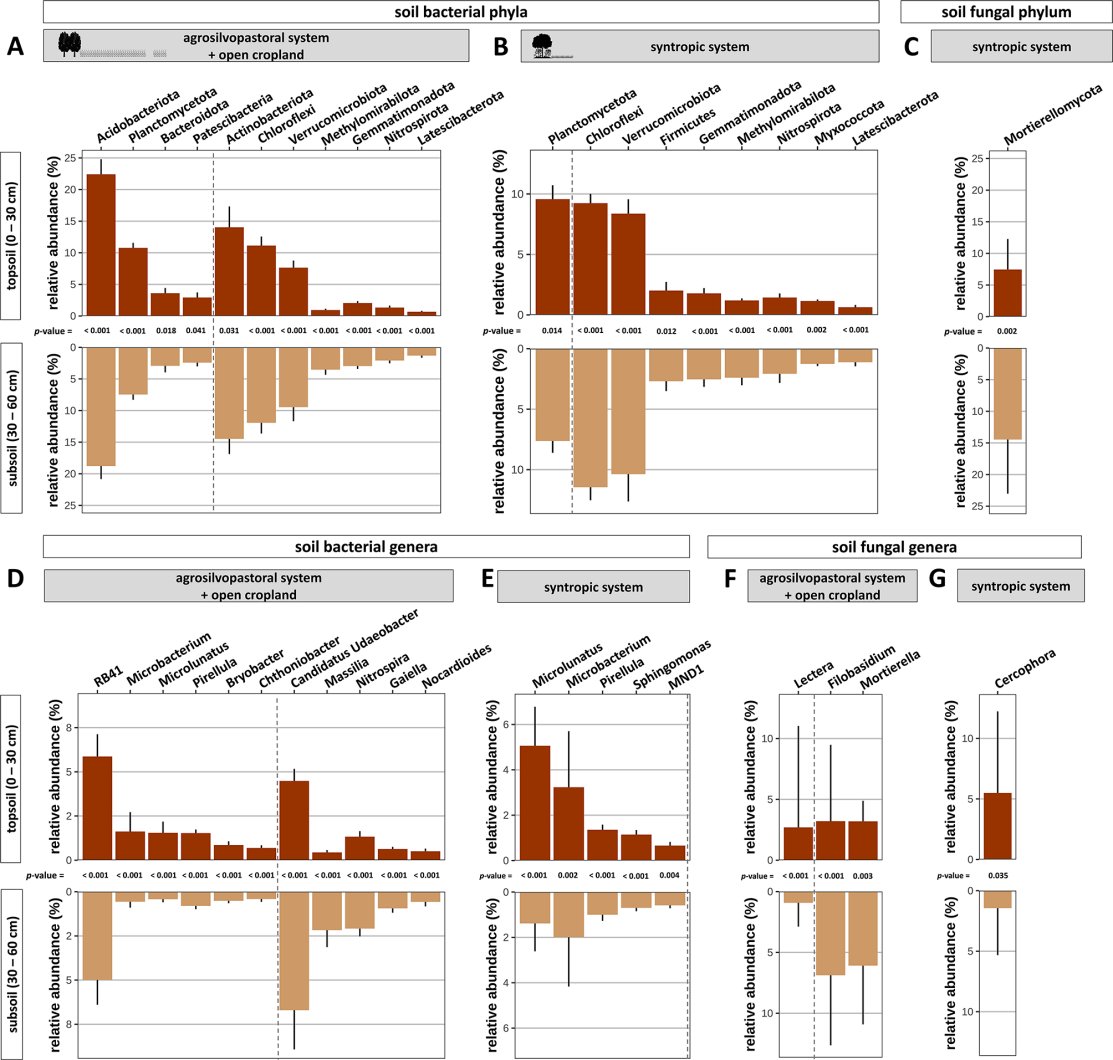
Differentially abundant soil bacterial (**A**, **B**, **D**, **E**) and fungal (**C**, **F**, **G**) phyla and genera in topsoil and subsoil of an agrosilvopastoral (*n* = 36) and syntropic alley cropping system (*n* = 12) near Alt Madlitz, Germany. Bars left from the dashed line show greater relative abundance in topsoil than subsoil, whereas bars right from the dashed line show greater relative abundance in subsoil than topsoil. The p-values between bars indicate statistically significant difference between topsoil and subsoil for the relative abundance of the respective phyla or genera. Statistically significant differences were determined using linear models for differential abundance analysis (LinDA) utilizing linear mixed-effect models with soil depth as a fixed effect and sampling location as random effect (*p* < 0.05). Icons were created with BioRender.com.

Soil depth strongly influenced the relative abundance of multiple bacterial groups in both agroforestry systems. In the agrosilvopastoral system, relative abundance of members of the bacterial phyla *Acidobacteriota*,* Bacteroidota*, *Patescibacteria*, and *Planctomycetota* were greater in topsoil than in subsoil (Fig. 6A). Likewise, members of the genera *Bryobacter*, *Chthoniobacter*,* Microbacterium*,* Microlunatus*,* Pirellula*, and *RB41* (*Acidobacteriota*) showed lower relative abundance in subsoil than topsoil of the same agroforestry system (Fig. 6D). Conversely, members of the phyla *Actinobacteriota*,* Chloroflexi*,* Gemmatimonadota*,* Latescibacterota*, *Methylomirabilota*,* Nitrospirota*, and *Verrucomicrobiota* and of the genera *Candidatus Udaeobacter* (*Verrucomicrobiota*), *Gaiella*, *Massilia*,* Nitrospira*, and *Nocardioides* showed greater relative abundance in subsoil of the agrosilvopastoral system than in topsoil (Fig. 6A, D). In the syntropic system, only the phylum *Planctomyctota* showed greater relative abundance in topsoil than subsoil, while the phyla *Chloroflexi*, *Firmicutes*, *Gemmatimonadota*, *Latescibacterota*,* Methylomirabilota*, *Myxococcota*,* Nitrospira*, and *Verrucomicrobiota* were promoted in subsoil (Fig. 6B). In contrast, on genus level, relative abundance of *Microlunatus*, *Microbacterium*, *MND1* (*Proteobacteria*), *Pirellula*, and *Sphingomonas* were greater in topsoil as compared to subsoil (Fig. 6E).

Within the fungal community of the agrosilvopastoral system, only the genera *Lectera* showed greater relative abundance in topsoil than subsoil, whereas members of *Filobasidium* and *Mortierella* were greater in subsoil than topsoil (Fig. 6F). Similarly, the phylum *Mortierellomycota* showed greater relative abundance in subsoil of the syntropic system than in topsoil (Fig. 6C), whereas the genus *Cercophora* showed the opposite pattern (Fig. 6G).

**Fig. 7:**
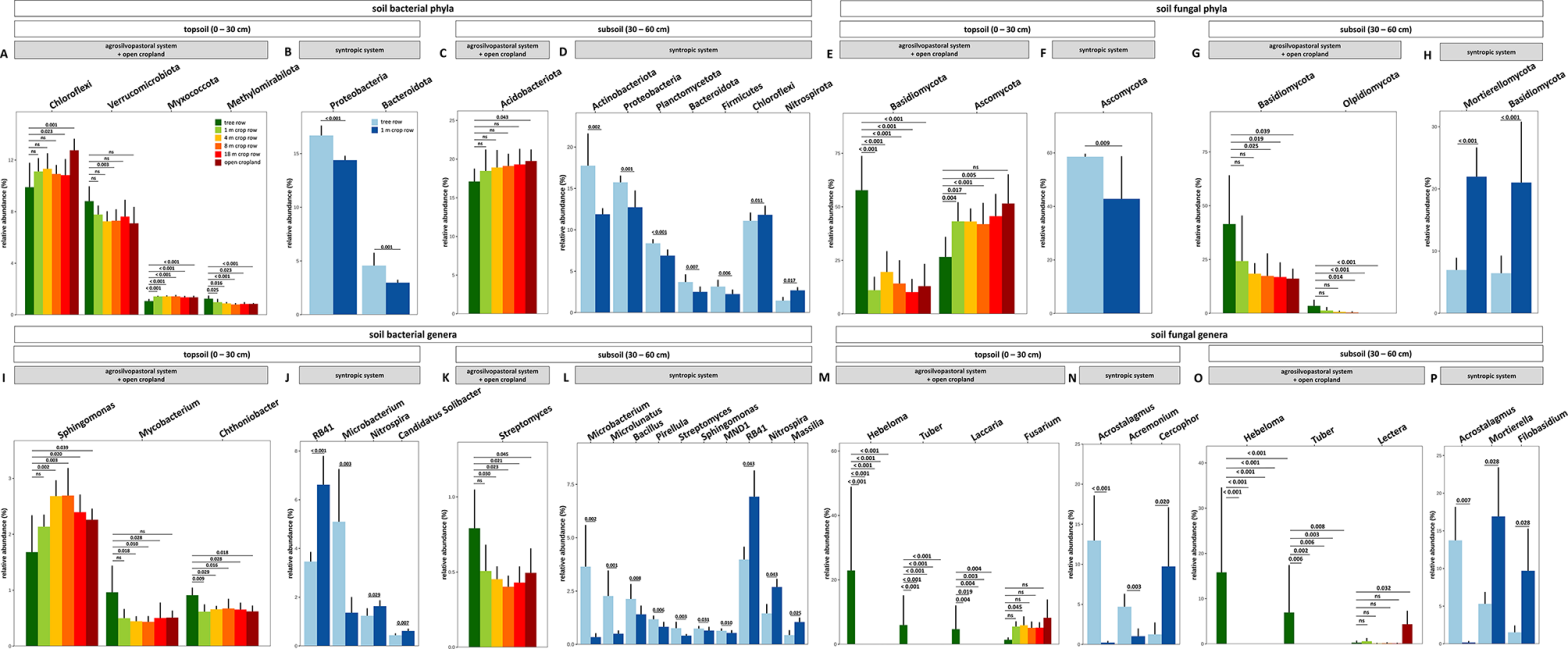
Relative abundance of bacterial (**A**,** B**,** C**,** D**,** I**,** J**,** K**,** L**) and fungal (**E**,** F**,** G**,** H**,** M**,** N**,** O**,** P**) phyla and genera in topsoil and subsoil of an agrosilvopastoral and syntropic alley cropping system near Alt Madlitz, Germany (*n* = 6). The p-values above bars indicate statistically significant difference between the tree row and the other sampling locations within the respective alley cropping system. Statistically significant differences were analysed using linear models for differential abundance analysis (LinDA) with tree row set as reference group for differential abundance analysis (*p* < 0.05).

In topsoil of the paired agrosilvopastoral and open cropland system, sampling location influenced the relative abundance of several bacterial phyla and genera (Fig. 7A, I). The relative abundance of the phyla *Methylomirabilota* and *Verrucomicrobiota* as well as the genera *Chthoniobacter* and *Mycobacterium* showed a pattern of greater relative abundance in the tree row as compared to the crop row and open cropland. Conversely, the relative abundance of the phyla *Chloroflexi* and *Myxococcota* were generally lower in the tree row than at sampling locations in the crop row and open cropland. Similarly, the genus *Sphingomonas* showed lower relative abundance under the trees of the agrosilvopastoral system as compared to all other sampling locations except for the distance closest to the trees in the crop row. In the syntropic system, however, only the phyla *Bacteroidota* and *Proteobacteria* were influenced by the sampling location, showing greater relative abundance in topsoil of the tree row as compared to the crop row (Fig. 7B). Similar results were obtained for the genus *Microbacterium*, whereas the genera *Candidatus Solibacter* (*Acidobacteriota*), *Nitrospira*, and *RB41* showed the opposite pattern of lower relative abundance under the trees than in the crop row (Fig. 7J).

In subsoil of the paired agrosilvopastoral and open cropland system, relative abundance of the phylum *Acidobacteriota* and the genus *Streptomyces* were affected by the trees (Fig. 7C, K). In the syntropic system, relative abundance of members of the phyla *Actinobacteriota*, *Bacteroidota*, *Firmicutes*,* Planctomycetota*, and *Proteobacteria* were greater in the tree row as compared to the crop row while *Chloroflexi* and *Nitrospira* showed the opposite trend (Fig. 7D). Similarly, members of the genera *Bacillus*,* Microbacterium*, *Microlunatus*, *MND1* (*Proteobacteria*), *Pirellula*, *Sphingomonas*, and *Streptomyces* were promoted under the trees, whereas, *Nitrospira*, *Massilia*, and *RB41* showed greater relative abundance in the crop row than in the tree row (Fig. 7L).

Sampling locations in both alley cropping systems contributed to differentially abundant fungal groups in topsoil and subsoil. In the paired agrosilvopastoral and open cropland system, relative abundance of the phylum *Basidiomycota* was greater under the trees than in the arable land, whereas the opposite pattern emerged for *Ascomycota* (Fig. 7E). In subsoil, relative abundance of *Basidiomycota* and *Olpidiomycota* gradually declined with increasing distance from the trees (Fig. 7G). *Ascomycota* and *Basidiomycota* showed opposing patterns in the syntropic system with greater abundance of *Ascomycota* and lower abundance of *Basidiomycota* in the tree row than in the crop row in topsoil and subsoil, respectively (Fig. 7F, H). In both topsoil and subsoil of the agrosilvopastoral system, relative abundance of *Hebeloma* and *Tuber* spp. were strongly increased under the trees as compared to the arable land, whereas relative abundance of *Lectera* spp. was greater in the open cropland than in the tree row (Fig. 7M, O). Sampling location further affected the relative abundance of *Fusarium* spp. but only in topsoil (Fig. 7M). In the syntropic system, relative abundance of *Agrostalagmus* spp. increased under the trees as compared to the crop row in both topsoil and subsoil, while *Agremonium* and *Cercophoria* spp. showed only effects in topsoil (Fig. 7N, P). In subsoil of the syntropic system, relative abundance of *Mortierella* and its phylum *Mortierellomycota* as well as *Filobasidium* were lower in the tree row than in the crop row (Fig. 7H, P).

## Discussion

Despite the large diversity of temperate alley cropping systems, so far most research focused on rather non-complex systems in terms of management and tree species composition. In this work, we investigated the topsoil and subsoil microbiome of two complex alley cropping systems, namely, an agrosilvopastoral and a syntropic system. Overall, we found that the richness of bacterial and fungal taxa declined from topsoil to subsoil in both agroforestry systems, which we attribute to resource-deprived conditions in subsoil. Accompanied with the decline in taxa richness, we detected a community shift for both bacterial and fungal communities from topsoil to subsoil. In both agroforestry systems, the tree rows harboured bacterial and fungal communities that were compositionally distinct from those in the crop rows. Furthermore, in the agrosilvopastoral system, the soil microbiome became compositionally less homogeneous with decreasing distance to the trees.

### Patterns of SOC in two young and differently managed alley cropping systems

SOC in topsoil and subsoil of the agrosilvopastoral system remained largely unaffected by the integration of trees (Fig S1A, B), which is likely due to the young age of the system (4 years). Indeed, recent literature suggests that increases in SOC stocks in alley cropping systems are usually detectable after one decade post their establishment^65^. In contrast, SOC under the trees in the syntropic system was greater than in the crop row although both systems are of the same age. The intensive mulching of tree rows most likely caused the rapid increase in SOC under the trees. Although we did not determine different SOC fractions, we expect that mainly labile SOC fractions increased through mulching as these fractions can respond rapidly to environmental changes. Furthermore, labile SOC fractions are considered indicators of long-term SOC trends^66^, however, their fast turnover and sensitivity towards environmental changes also makes them vulnerable to loss^67^. In a case study from 2015, Cardinael and others^68^ suggested that the additional SOC storage under the trees of an 18-year-old walnut alley cropping system in France was mostly due to an increase in labile SOC fractions, raising questions regarding its stability. We therefore suggest that future research on SOC stocks in alley cropping systems should consider differentiating between labile and stable SOC fractions in order to infer information on SOC stabilization dynamics in alley cropping systems.

### Soil depth as a key driver of the abundance and community composition of bacteria and fungi

Soil depth is a key driver of the assembly of the soil microbiome since multiple influencing factors such as the availability of oxygen and other resources generally decrease as soil depth increases. The scarcity of resources in subsoil serves as a reasonable explanation for the observed decline in population size of bacteria, fungi, and archaea with increasing soil depth (Fig. 2) that confirms our first hypothesis and is congruent with previous studies^69–72^. Furthermore, specialization to resource-deprived conditions in subsoil also explains the decline in bacterial species richness from topsoil to subsoil of the agrosilvopastoral system (Fig. 3) as only a limited number of taxa can thrive in subsoil^72^.

In addition to changes in alpha diversity, soil depth led to compositionally distinct bacterial communities in both agroforestry systems. For instance, multiple phyla associated with an oligotroph lifestyle such as *Chloroflexi*, *Verrucomicrobiota*, *Gemmatimonadota*, *Nitrospirota*, and *Latescibacterota* showed greater relative abundance in subsoil than topsoil, highlighting their ability to adapt to resource-deprived soil environments. Similarly, in the agrosilvopastoral system, *Gaiella* and *Nocardioides* spp. as well as their affiliated phylum *Actinobacteria* showed a similar pattern of promotion in subsoil, which is in line with their presumed oligotrophic lifestyle. However, it should be noted that the generalized assignment of a single lifestyle to a broad taxonomic group may not match the lifestyles of all its members^73,74^. Although our results for certain phyla mentioned above (*Actinoabacteria*, *Chloroflexi*, *Nitrospirota*, and *Latescibacterota*) are in line with previous studies that investigated bacterial communities at different soil depths^69,75^, Lopes et al.^76^ reported opposing results for *Verrucomicrobiota* and *Gemmatimonadota* (i.e. greater relative abundance in topsoil than subsoil). For instance, the authors found the genus *Candidatus Udaeobacter* (*Verrucomicrobiota*) to be depleted in deeper soil layers and attributed this finding to its aerobic lifestyle^76,77^. However, in the agrosilvopastoral system, relative abundance of *Candidatus Udaeobacter* was greater in subsoil than topsoil (Fig. 6D). Such a discrepancy may partly be due to differences in sampling depth: while Lopes and co-authors^76^ sampled up to 240 cm deep, subsoil samples in our study were collected at 30–60 cm depth, which likely did not cover anaerobic conditions. Likewise, in 2015, Navarette et al.^78^ reported decreasing relative abundance of *Verrucomicrobiota* with increasing soil fertility, which coincides with the observed increased proportion of this phylum in resource-deprived subsoil (Fig. 6A, B).

In contrast to bacteria, only very few fungal phyla and genera were differentially abundant between topsoil and subsoil; however, in the agrosilvopastoral and syntropic system, members of *Mortierellomycota* and *Mortierella* showed greater relative abundance in subsoil than topsoil, respectively (Fig. 6C, F). Although information on the vertical distribution of *Mortierellomycota* and *Mortierella* spp. in agricultural soils is scarce, *Mortierella* spp. are widely recognized as important decomposers as well as for their benefits as plant-growth-promoting fungi^79,80^. Another fungal genus that was dominant in subsoil of the agrosilvopastoral system was *Filobasidium* (Fig. 6F). The genus *Filobasidium*harbours members with the ability to produce extracellular polymeric substances, which enable them to form biofilms^81^, a survival mechanism by which these organisms may cope with the resource-deprived conditions in subsoil.

In addition to a distinct community composition between soil depths, community similarity of bacteria and fungi was greater within topsoil than subsoil (Fig. 5), revealing greater compositional homogenization of the topsoil than the subsoil microbiome and thereby confirming our third hypothesis. As our study sites are located in a glacial landscape that is characterized by small-scale spatial variability of soil properties, we expected a comparatively large dissimilarity in microbiome composition among samples. Although our study sites were managed under reduced tillage for five years, the previous decade-long tillage regime likely reduced the degree of spatial variability of soil properties in topsoil through mechanical homogenization while subsoil remained undisturbed. We therefore propose that spatial homogenization of topsoil through tillage translated into compositional homogenization of topsoil biota. Likewise, we argue that greater spatial variability of soil properties in deeper soil layers contributed to greater community dissimilarity in subsoil as compared to topsoil.

### Distance to trees shapes the soil microbiome in agroforestry systems

The distance to the tree rows influenced bacterial and fungal community composition in both systems. For bacterial communities, most striking effects of the trees were observed in subsoil of the syntropic system: *Actinobacteria* and its affiliated genera *Microbacterium*,* Microlunatus*, and *Streptomyces* showed greater relative abundance under the trees than in the crop row. Members of these groups are often found in rhizosphere and include species that are recognized for their antimicrobial properties as well as their plant growth promoting effects^82,83^. We found similar effects for *Proteobacteria* and its affiliated genera *Sphingomonas* and *MND1* that are also frequently recovered from rhizosphere with some strains showing beneficial effects on plant growth^84–86^. The syntropic system is characterized by high diversity of plants, which may explain the promotion of putative beneficial bacteria that are known for root interactions. We expect the aboveground plant diversity and density to be mirrored belowground through the formation of a complex rooting system along the soil profile that creates numerous habitats for soil organisms. In line with this assumption, we observed greater mean population size of bacteria, fungi, and archaea in the tree row than the crop row of the syntropic system, partly confirming our second hypothesis (Fig. 2). The promotion of microbial population size was likely due to increased root biomass and root exudates as plant diversity increased as shown previously for bacteria and fungi in a microcosm experiment^35^.

In topsoil of the agrosilvopastoral system, trees specifically promoted ectomycorrhizal fungi (EMF) of the genera *Hebeloma*, *Laccaria*, and *Tuber* (Fig. 7M). This finding is not surprising considering the symbiotic association that EMF form with poplar trees and agrees with our previous findings^87^. Furthermore, for *Hebeloma* and *Tuber* spp., the promotion through the trees was also detected in subsoil, indicating that the formation of beneficial tree–microbe relationships extended into deeper soil layers. In contrast, the genus *Fusarium*, which harbours numerous economically relevant plant pathogens, showed a pattern of lower relative abundance under the trees than in the crop row and open cropland in topsoil. While this pattern could simply emerge due to the absence of their host plants in the tree rows, increased microbial antagonism as well as enhanced biological control through improved soil faunal activity under the trees as proposed by Vaupel et al.^87^ may have contributed to the observed pattern. Together with our results on potentially beneficial bacteria and EMF, lower abundance of *Fusarium* spp. under the trees than the crops confirms our fourth hypothesis that trees promote beneficial and symbiotic microorganisms rather than putative phytopathogens.

The introduction of tree rows in arable land through agroforestry is frequently described as a diversification measure that increases spatial heterogeneity by enabling biological interactions between trees and crops^88^. Consequently, biological interactions within alley cropping systems can be expected to be spatially depended on the distance from the trees into the crop rows. In order to capture such spatial heterogeneity, sampling soil along linear transects spanning from the tree rows into the crop rows is advisable. Using transect sampling within the agrosilvopastoral systems, we here for the first time report that (i) tree rows in a temperate agroforestry system not only promote microbial community dissimilarity in both topsoil and subsoil but that (ii) community dissimilarity decreased with increasing distance from the trees into the crop rows and the open cropland (Fig. 5). In other words, compared to the open cropland system, spatial heterogeneity introduced by the tree rows in the agrosilvopastoral system translated into a compositionally less homogeneous soil microbiome that became increasingly homogenous as the distance from the trees increased. However, we did not observe this pattern in the syntropic system, which we expect to be due to the intensive mulching of the tree rows that has a strong impact on soil microbial communities^89,90^. Nevertheless, our finding on community dissimilarity in the agrosilvopastoral system is of particular interest given that land-use change from natural ecosystems to agriculture has recently been shown to result in taxonomic homogenization of soil microorganisms^91^. Agroforestry practice may counteract the homogenization of the soil microbiome through agriculture.

## Conclusion

Soil depth strongly affected bacterial and fungal richness and community composition in both the agrosilvopastoral and syntropic agroforestry system. In addition to a decline in microbial richness from topsoil to subsoil, we observed a community shift towards oligotrophic microorganisms with increasing soil depth, as only a limited number of taxa can thrive in the resource-deprived conditions in subsoil. At both soil depths, tree rows of both systems harboured compositionally distinct microbiomes from those of the crop rows. The soil microbiome modulated by the trees showed a shift towards beneficial microorganisms (potential plant growth-promoting bacteria and EMF) while putative phytopathogens were found in lower relative abundance as compared to the crop rows. Finally, we show that compared to an open cropland system without trees, spatial heterogeneity introduced by the tree rows in the agrosilvopastoral system translated into a compositionally less homogeneous soil microbiome that became increasingly homogenous as the distance from the trees increased. Therefore, we suggest that agroforestry can counteract the homogenization of the soil microbiome through agriculture.

## Electronic supplementary material

Below is the link to the electronic supplementary material.

Supplementary Material 1

Supplementary Material 2

## Data Availability

Amplicon sequencing data have been deposited at NCBI’s Short Read Archive (BioProject https://dataview.ncbi.nlm.nih.gov/object/PRJNA1142703?reviewer=8tmcmrc85peublr8q712s4immh for bacteria and https://dataview.ncbi.nlm.nih.gov/object/PRJNA1142770?reviewer=ss0detkc3eqfdj833q4rs2nbs8 for fungi).
