# Supplementary material for: Trees shape the soil microbiome of a temperate agrosilvopastoral and syntropic agroforestry system
Source: Sci Rep. 2025 Jan 9;15:1550. doi: 10.1038/s41598-025-85556-4 (PMC11717919; doi:10.1038/s41598-025-85556-4)
Supplement: Supplementary file 1 — Supplementary Material 1 [file 41598_2025_85556_MOESM1_ESM.pdf]

Table S1. Results of permutational multivariate analysis of variance (PERMANOVA) based on soil bacterial ASV counts using 999 permutations for different soil depths in the agrosilvopastoral and syntropic alley-cropping system

agrosilvopastoral agroforestry + open cropland					
	Df	Sum sqs	R^2	F	p-value
**soil depth**[a]	1	3.6748	0.2225	20.032	**0.001**
**residual**	70	12.8416	0.7775		
**total**	71	16.5164	1.0		

syntropic agroforestry					
	Df	Sum sqs	R^2	F	p-value
**soil depth**[a]	1	0.7483	0.1658	4.3731	**0.001**
**residual**	10	3.7643	0.8342		
**total**	11	4.5125	1.0		

Df = degrees of freedom; Sum Sqs = sum of squares; R^2 = coefficient of determination; F = pseudo − F ratio; p-values indicating significant differences are printed in bold

[a] = sampling depth of topsoil (0-30 cm) and subsoil (30-60 cm)

Table S2. Results of permutational multivariate analysis of variance (PERMANOVA) based on soil bacterial ASV counts using 999 permutations for different sampling locations in topsoil and subsoil of the agrosilvopastoral alley-cropping system

agrosilvopastoral agroforestry + open cropland topsoil					
	Df	Sum sqs	R^2	F	p-value
**sampling location**[a]	5	1.1809	0.2308	1.7999	**0.001**
**residual**	30	3.9368	0.7693		
**total**	35	5.1177	1.0		

agrosilvopastoral agroforestry + open cropland subsoil					
	Df	Sum sqs	R^2	F	p-value
**sampling location**[a]	5	1.4167	0.1834	1.3477	**0.001**
**residual**	30	6.3072	0.8166		
**total**	35	7.7238	1.0		

Df = degrees of freedom; Sum Sqs = sum of squares; R^2 = coefficient of determination; F = pseudo − F ratio; p-values indicating significant differences are printed in bold

[a] = sampling locations in the agrosilvopastoral (tree row, 1, 4, 8, and 18 m crop row and open cropland) and syntropic (tree row, 5 m crop row) alley-cropping system

Table S3. Results of permutational multivariate analysis of variance (PERMANOVA) based on soil bacterial ASV counts using 999 permutations for different sampling locations in topsoil and subsoil of the syntropic alley-cropping system

syntropic agroforestry topsoil					
	**Df**	**Sum sqs**	***R^2***	***F***	**p-value**
**sampling location[a]**	1	0.3209	0.2054	2.5847	**0.001**
**residual**	10	1.2414	0.7946		
**total**	11	1.5622	1.0		

syntropic agroforestry subsoil					
	**Df**	**Sum sqs**	***R^2***	***F***	**p-value**
**sampling location[a]**	1	0.4080	0.1853	2.2738	**0.003**
**residual**	10	1.7941	0.8148		
**total**	11	2.2021	1.0		

Df = degrees of freedom; Sum Sqs = sum of squares; R^2 = coefficient of determination; F = pseudo − F ratio; p-values indicating significant differences are printed in bold

[a] = sampling locations in the agrosilvopastoral (tree row, 1, 4, 8, and 18 m crop row and open cropland) and syntropic (tree row, 5 m crop row) alley-cropping system

Table S4. Results of the pairwise permutational multivariate analysis of variance (PERMANOVA) based on soil bacterial ASV counts using 999 permutations for different sampling locations in topsoil and subsoil of the agrosilvopastoral alley-cropping system

**agrosilvopastoral agroforestry + open cropland topsoil**				
**Pairwise comparison**	***F***	***R²***	**p-value unadj.**	**p-value adj.**
**tree row vs. 1 m crop row**	1.4969	0.1302	0.016	**0.0240**
**tree row vs. 4 m crop row**	1.8200	0.1540	0.004	**0.0075**
**tree row vs. 8 m crop row**	2.1371	0.1761	0.003	**0.0075**
**tree row vs. 18 m crop row**	1.9387	0.1624	0.003	**0.0075**
**tree row vs. open cropland**	2.5941	0.2060	0.004	**0.0075**
**1 m crop row vs. 4 m crop row**	0.8054	0.0745	0.877	0.8770
**1 m crop row vs. 8 m crop row**	1.2475	0.1109	0.099	0.1325
**1 m crop row vs. 18 m crop row**	1.1557	0.1036	0.14	0.1500
**1 m crop row vs. open cropland**	2.2497	0.1837	0.004	**0.0075**
**4 m crop row vs. 8 m crop row**	1.2621	0.1121	0.106	0.1325
**4 m crop row vs. 18 m crop row**	1.1324	0.1017	0.128	0.1477
**4 m crop row vs. open cropland**	2.1927	0.1798	0.005	**0.0083**
**8 m crop row vs. 18 m crop row**	1.7180	0.1466	0.004	**0.0075**
**8 m crop row vs. open cropland**	3.2749	0.2467	0.002	**0.0075**
**18 m crop row vs. open cropland**	2.2027	0.1805	0.003	**0.0075**

**agrosilvopastoral agroforestry + open cropland subsoil**				
**Pairwise comparison**	***F***	***R²***	**p-value unadj.**	**p-value adj.**
**tree row vs. 1 m crop row**	1.0470	0.0948	0.311	0.4241
**tree row vs. 4 m crop row**	1.1581	0.1038	0.168	0.2800
**tree row vs. 8 m crop row**	1.6647	0.1427	0.02	0.0500
**tree row vs. 18 m crop row**	1.4509	0.1267	0.039	0.0788
**tree row vs. open cropland**	1.8628	0.1570	0.017	0.0500
**1 m crop row vs. 4 m crop row**	0.7994	0.0740	0.912	0.9120
**1 m crop row vs. 8 m crop row**	1.1157	0.1004	0.22	0.3300
**1 m crop row vs. 18 m crop row**	0.8669	0.0798	0.854	0.9120
**1 m crop row vs. open cropland**	1.7145	0.1464	0.011	**0.0413**
**4 m crop row vs. 8 m crop row**	0.8861	0.0814	0.751	0.8665
**4 m crop row vs. 18 m crop row**	1.0098	0.0917	0.407	0.5088
**4 m crop row vs. open cropland**	1.7095	0.1460	0.011	**0.0413**
**8 m crop row vs. 18 m crop row**	1.2587	0.1118	0.042	0.0788
**8 m crop row vs. open cropland**	1.9677	0.1644	0.004	**0.0300**
**18 m crop row vs. open cropland**	1.8136	0.1535	0.003	**0.0300**

Df = degrees of freedom; Sum Sqs = sum of squares; R^2 = coefficient of determination; F = pseudo − F ratio; p-values indicating significant differences are printed in bold

Table S5. Results of permutational multivariate analysis of variance (PERMANOVA) based on soil fungal ASV counts using 999 permutations for different soil depths in the agrosilvopastoral and syntropic alley-cropping system

agrosilvopastoral agroforestry + open cropland					
	**Df**	**Sum sqs**	***R^2***	***F***	**p-value**
**soil depth[a]**	1	1.453	0.0746	5.6388	**0.001**
**residual**	70	18.038	0.9255		
**total**	71	19.491	1.0		

syntropic agroforestry					
	**Df**	**Sum sqs**	***R^2***	***F***	**p-value**
**soil depth[a]**	1	0.3079	0.0530	1.2299	0.215
**residual**	10	5.5070	0.9471		
**total**	11	5.8148	1.0		

Df = degrees of freedom; Sum Sqs = sum of squares; R^2 = coefficient of determination; F = pseudo − F ratio; p-values indicating significant differences are printed in bold

[a] = sampling depth of topsoil (0-30 cm) and subsoil (30-60 cm)

Table S6. Results of permutational multivariate analysis of variance (PERMANOVA) based on soil fungal ASV counts using 999 permutations for different sampling locations in topsoil and subsoil of the agrosilvopastoral alley-cropping system

agrosilvopastoral agroforestry + open cropland topsoil					
	**Df**	**Sum sqs**	***R^2***	***F***	**p-value**
**sampling location[a]**	5	2.4345	0.3055	2.6396	**0.001**
**residual**	30	5.5339	0.6945		
**total**	35	7.9685	1.0		

agrosilvopastoral agroforestry + open cropland subsoil					
	**Df**	**Sum sqs**	***R^2***	***F***	**p-value**
**sampling location[a]**	5	2.1377	0.2123	1.617	**0.001**
**residual**	30	7.9320	0.7877		
**total**	35	10.0697	1.0		

Df = degrees of freedom; Sum Sqs = sum of squares; R^2 = coefficient of determination; F = pseudo − F ratio; p-values indicating significant differences are printed in bold

[a] = sampling locations in the agrosilvopastoral (tree row, 1, 4, 8, and 18 m crop row and open cropland) and syntropic (tree row, 5 m crop row) alley-cropping system

Table S7. Results of permutational multivariate analysis of variance (PERMANOVA) based on soil fungal ASV counts using 999 permutations for different sampling locations in topsoil and subsoil of the syntropic alley-cropping system

		**syntropic agroforestry topsoil**			
	**Df**	**Sum sqs**	***R²***	***F***	**p-value**
**sampling location**[a]	1	0.720	0.2669	3.6404	**0.003**
**residual**	10	1.9778	0.7331		
**total**	11	2.6978	1.0		

		**syntropic agroforestry subsoil**			
	**Df**	**Sum sqs**	***R²***	***F***	**p-value**
**sampling location**[a]	1	0.9944	0.3540	5.4796	**0.002**
**residual**	10	1.8148	0.6460		
**total**	11	2.8092	1.0		

Df = degrees of freedom; Sum Sqs = sum of squares; R^2 = coefficient of determination; F = pseudo − F ratio; p-values indicating significant differences are printed in bold

[a] = sampling locations in the agrosilvopastoral (tree row, 1, 4, 8, and 18 m crop row and open cropland) and syntropic (tree row, 5 m crop row) alley-cropping system

Table S8. Results of the pairwise permutational multivariate analysis of variance (PERMANOVA) based on soil fungal ASV counts using 999 permutations for different sampling locations in topsoil and subsoil of the agrosilvopastoral alley-cropping system

agrosilvopastoral agroforestry + open cropland topsoil				
**Pairwise comparison**	***F***	***R^2***	**p-value unadj.**	**p-value adj.**
**tree row vs. 1 m crop row**	3.1969	0.2422	0.003	**0.0075**
**tree row vs. 4 m crop row**	2.8704	0.2230	0.001	**0.0050**
**tree row vs. 8 m crop row**	3.4656	0.2574	0.002	**0.0060**
**tree row vs. 18 m crop row**	3.1228	0.2380	0.001	**0.0050**
**tree row vs. open cropland**	3.6211	0.2658	0.001	**0.0050**
**1 m crop row vs. 4 m crop row**	0.7701	0.0715	0.876	0.8760
**1 m crop row vs. 8 m crop row**	0.8517	0.0785	0.706	0.8146
**1 m crop row vs. 18 m crop row**	0.9006	0.0826	0.658	0.8146
**1 m crop row vs. open cropland**	3.9235	0.2818	0.005	**0.0083**
**4 m crop row vs. 8 m crop row**	0.8173	0.0756	0.79	0.8464
**4 m crop row vs. 18 m crop row**	0.9615	0.0877	0.548	0.7473
**4 m crop row vs. open cropland**	3.6297	0.2663	0.004	**0.0075**
**8 m crop row vs. 18 m crop row**	1.0066	0.0915	0.464	0.6960
**8 m crop row vs. open cropland**	4.9841	0.3326	0.002	**0.0060**
**18 m crop row vs. open cropland**	3.6748	0.2687	0.004	**0.0075**

agrosilvopastoral agroforestry + open cropland subsoil				
**Pairwise comparison**	***F***	***R^2***	**p-value unadj.**	**p-value adj.**
**tree row vs. 1 m crop row**	1.3715	0.1206	0.072	0.1200
**tree row vs. 4 m crop row**	2.0275	0.1686	0.016	**0.0300**
**tree row vs. 8 m crop row**	1.9060	0.1601	0.008	**0.0200**
**tree row vs. 18 m crop row**	2.0750	0.1718	0.011	**0.0236**
**tree row vs. open cropland**	2.6636	0.2103	0.004	**0.0188**
**1 m crop row vs. 4 m crop row**	0.8901	0.0817	0.718	0.8285
**1 m crop row vs. 8 m crop row**	0.7661	0.0712	0.967	0.9670
**1 m crop row vs. 18 m crop row**	0.9774	0.0890	0.481	0.7215
**1 m crop row vs. open cropland**	2.1400	0.1763	0.007	**0.0200**
**4 m crop row vs. 8 m crop row**	0.9161	0.0839	0.691	0.8285
**4 m crop row vs. 18 m crop row**	0.9161	0.0839	0.596	0.8127
**4 m crop row vs. open cropland**	2.3215	0.1884	0.005	**0.0188**
**8 m crop row vs. 18 m crop row**	0.8220	0.0760	0.782	0.8379
**8 m crop row vs. open cropland**	2.3156	0.1880	0.002	**0.0188**
**18 m crop row vs. open cropland**	2.3022	0.1871	0.003	**0.0188**

Df = degrees of freedom; Sum Sqs = sum of squares; R^2 = coefficient of determination; F = pseudo − F ratio; p-values indicating significant differences are printed in bold
