# Supplementary material for: Trees shape the soil microbiome of a temperate agrosilvopastoral and syntropic agroforestry system
Source: Sci Rep. 2025 Jan 9;15:1550. doi: 10.1038/s41598-025-85556-4 (PMC11717919; doi:10.1038/s41598-025-85556-4)
Supplement: Supplementary file 2 — Supplementary Material 2 [file 41598_2025_85556_MOESM2_ESM.pdf]

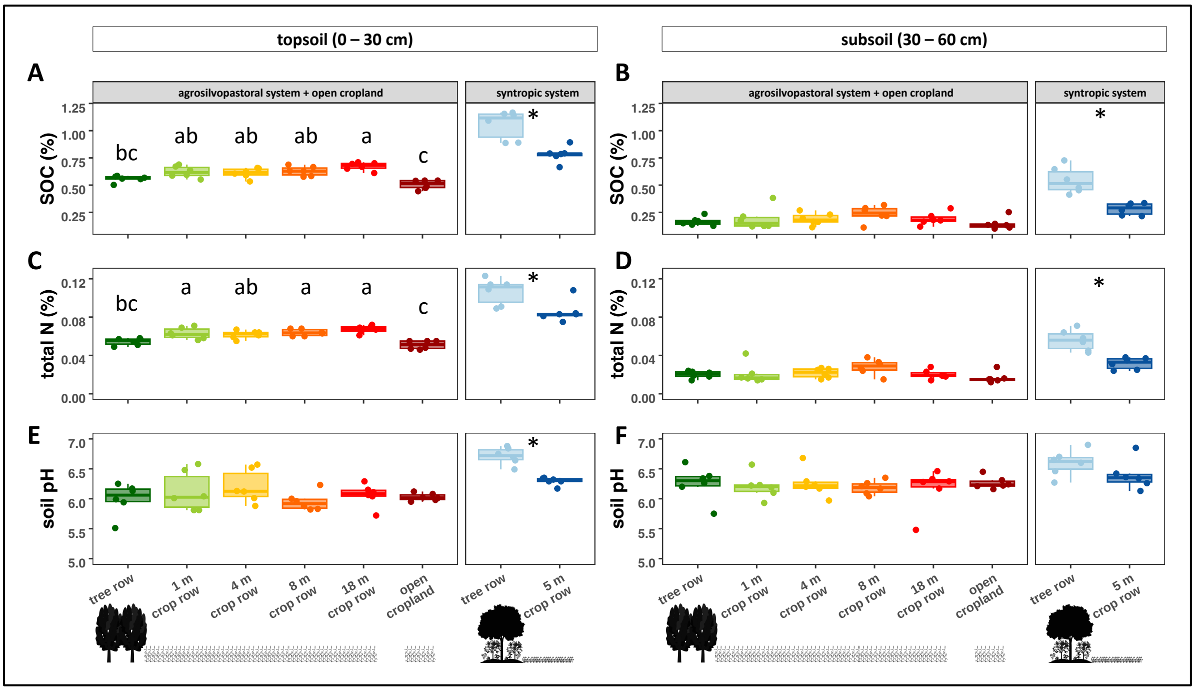

Supplementary Figure S1. General soil properties (soil organic C (SOC) (**A**, **B**), total N (**C**, **D**), and soil pH (**E**, **F**)) in topsoil and subsoil of an agrosilvopastoral and syntropic alley-cropping system near Alt Madlitz, Germany (n = 6). Dots indicate individual data points. Different letters indicate statistically significant differences among the six sampling locations within the agrosilvopastoral alley-cropping system (ANOVA followed by Tukey's HSD test; p ≤ 0.05). Asterisks indicate statistically significant differences between the two sampling locations within the syntropic alley-cropping system (Student's t-test p ≤ 0.05). Absence of letters or asterisks indicates no statistically significant differences among sampling locations (p > 0.05). Icons were created with BioRender.com

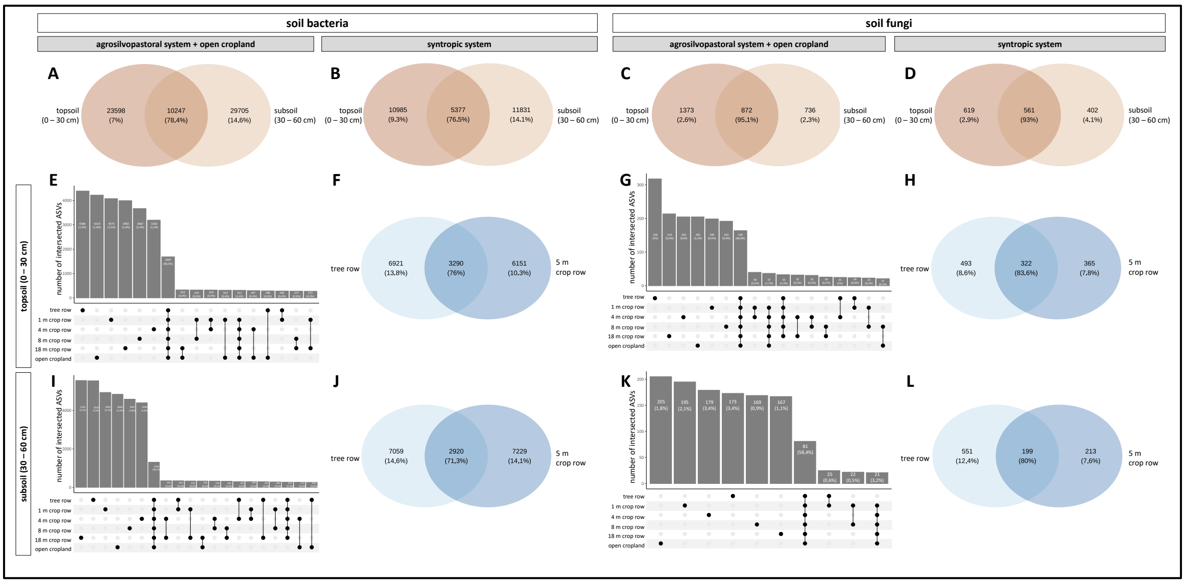

Supplementary Figure S2. Number of unique and shared amplicon sequencing variants (ASVs) (numbers in brackets indicate the percentage of total ASV counts) between sampling depths (**A, B, C, D**) of an agrosilvopastoral (n = 36) and syntropic alley-cropping system (n = 12) as well as between sampling locations in topsoil (**E, F, G, H**) and subsoil (**I, J, K, L**) of both systems (n = 6) near Alt Madlitz, Germany.
